# SmartBoot: Real-Time Monitoring of Patient Activity via Remote Edge Computing Technologies

**DOI:** 10.3390/s25144490

**Published:** 2025-07-19

**Authors:** Gozde Cay, Myeounggon Lee, David G. Armstrong, Bijan Najafi

**Affiliations:** 1 Digital Health and Access Center (DiHAC), Michael E. DeBakey Department of Surgery, Baylor College of Medicine, Houston, TX 77030, USA; gozde.cay@ikc.edu.tr (G.C.); myeounggon.lee@gmail.com (M.L.); 2 Department of Computer Engineering, Izmir Katip Celebi University, Izmir 35620, Türkiye; 3 Center for Advanced Surgical & Interventional Technology (CASIT), Department of Surgery, University of California, Los Angeles, CA 90095, USA; 4 Institute on Aging, Seoul National University, Seoul 01000, Republic of Korea; 5 Southwestern Academic Limb Salvage Alliance (SALSA), Department of Surgery, Keck School of Medicine, University of Southern California, Los Angeles, CA 90089, USA

**Keywords:** offloading, adherence, diabetic foot ulcer, wound care, telemedicine, digital health, remote patient monitoring, wearables, smart footwear, personalized care, edge computing

## Abstract

Diabetic foot ulcers (DFUs) are a serious complication of diabetes, associated with high recurrence and amputation rates. Adherence to offloading devices is critical for wound healing but remains inadequately monitored in real-world settings. This study evaluates the SmartBoot edge-computing system—a wearable, real-time remote monitoring solution integrating an inertial measurement unit (Sensoria Core) and smartwatch—for its validity in quantifying cadence and step count as digital biomarkers of frailty, and for detecting adherence. Twelve healthy adults wore two types of removable offloading boots (Össur and Foot Defender) during walking tasks at varied speeds; system outputs were validated against a gold-standard wearable and compared with staff-recorded adherence logs. Additionally, user experience was assessed using the Technology Acceptance Model (TAM) in healthy participants (n = 12) and patients with DFU (n = 81). The SmartBoot demonstrated high accuracy in cadence and step count across conditions (bias < 5.5%), with an adherence detection accuracy of 96% (Össur) and 97% (Foot Defender). TAM results indicated strong user acceptance and perceived ease of use across both cohorts. These findings support the SmartBoot system’s potential as a valid, scalable solution for real-time remote monitoring of adherence and mobility in DFU management. Further clinical validation in ongoing studies involving DFU patients is underway.

## 1. Introduction

Diabetic foot ulcers (DFUs) constitute one of the most severe complications of diabetes [1,2], primarily induced by peripheral neuropathy, known as “loss of protective sensation” or “absence of gifted pain” [3]. Globally, approximately 26.1 million individuals, or 2–3% of people with diabetes, develop DFUs annually, with a lifetime incidence rate of 15% [2]. Shockingly, a new ulcer develops worldwide every 1.2 s, leading to a lower limb amputation every 20 s in DFU patients [2]. The healthcare costs linked to DFUs are staggering, with acute care treatment expenses soaring to as much as USD 70,000 per event, dependent on the wound’s severity [4]. In the United States, approximately one-third of all diabetes-related costs are allocated to diabetic foot care, imposing a substantial financial burden on society [5,6]. The alarming 10% rate of lower extremity amputation associated with DFUs is particularly distressing, as nearly 70% of these amputations are potentially preventable [2]. This highlights the urgent need for effective DFU management and prevention, particularly among underserved populations and individuals residing in remote areas with limited healthcare access. For instance, research in Texas revealed that living more than 50 miles from a specialized hospital is a significant predictor of lower extremity amputation in individuals with diabetic foot syndrome [4]. Despite efforts, DFU management remains a challenging goal. In the absence of a definitive cure, it is paramount to reduce diabetes-related complications to enhance the health and quality of life for people with diabetes.

Contemporary approaches to the treatment and management of DFUs predominantly rely on the clinical expertise of podiatrists, with a primary focus on wound care and the implementation of offloading to alleviate pressure on affected areas. There are four offloading intervention categories: (1) offloading devices (removable or non-removable and ankle-high or knee-high devices); (2) footwear (shoes, insoles, socks, etc.); (3) surgery (silicone injections, tissue augmentation, tenotomy, etc.); and (4) other offloading techniques (bed rest, wheelchairs, braces, etc.). Offloading devices relieve and redistribute the mechanical stresses of plantar pressure (acting normal to the skin surface) and shear stresses (acting tangential to the skin surface) during weight-bearing activities [7]. It has been well established in research that the strict adherence of patients to prescribed offloading devices is pivotal for the successful treatment of DFUs [8,9,10,11,12,13]. Due to the importance of strict adherence, non-removable offloading devices are accepted as a gold standard to treat DFUs [7,14]. However, non-removable offloading devices are generally not preferred by patients with DFUs due to several limitations. These include low patient acceptance, limited prescription of the offloading devices; unsatisfactory patient-centered outcomes such as poor sleep quality; and a high risk of frailty or leg muscle atrophy, which in turn contributes to a high recurrence rate of DFUs [15,16,17,18,19]. As an alternative, removable offloading devices are proposed to the patients. Recent research shows that the removable offloading devices can also provide a similar healing rate to that of the non-removable devices if the patients follow the same adherence protocols [20,21]. This also highlights the importance of ensuring patient adherence with offloading devices.

Previously, we introduced the SmartBoot system—a sensor-based offloading device designed to monitor adherence in real-time and promote compliance through gamification features displayed on a low-cost smartwatch (e.g., a “happy face” when the boot is worn during weight-bearing, and a “sad face” when it is not) [11,12]. We demonstrated its acceptability among individuals with diabetic foot ulcers (DFUs) across diverse age groups and ethnic backgrounds [22]. In addition to adherence monitoring, SmartBoot captures digital biomarkers relevant to wound healing—such as daily steps (protected vs. unprotected) and cadence (a proxy for frailty)—and streams this data to the cloud to support remote patient monitoring and personalized care plans [23]. However, prior studies have not established the validity and reliability of SmartBoot for accurate, real-time measurement of steps, cadence, and adherence across different walking speeds and offloading modalities. To address this gap, we assessed SmartBoot’s accuracy among healthy individuals (to avoid ethical concerns in high-risk DFU populations) wearing two standard offloading devices—at knee level (DH Walker by Össur) and above the ankle (Foot Defender)—at varying walking speeds (slow, self-selected or habitual, and fast). To evaluate perceived usability and acceptability, we also recruited individuals with DFUs who used the SmartBoot during their wound care. Our hypotheses are as follows:

- Regardless of the boot type, the SmartBoot system can objectively measure adherence and weight-bearing activities, including cadence and step count.
- User perspectives are similar across both boot types, though patients may prefer the boot that is easier to use.

## 2. Materials and Methods

### 2.1. SmartBoot

The SmartBoot system and its data analysis method for remote and real-time extraction of the adherence, cadence, and number of steps were previously explained in our preliminary study [11]. Briefly, the SmartBoot is an advanced offloading system designed to enhance DFU management by integrating multiple components for real-time monitoring and feedback. It comprises a removable offloading boot, such as the Foot Defender, equipped with the Sensoria Core (Sensoria Health Inc., Redmond, WA, USA)—a microelectronic unit featuring a six-degree-of-freedom inertial measurement unit (IMU). Bluetooth low-energy (BLE) connectivity data is processed locally via a smartwatch acting as an edge computing hub, enabling on-device detection of inadequate offloading and calculation of key parameters, such as daily step count (protected vs. unprotected) and cadence. This setup enables immediate visual feedback to patients, reinforcing proper usage behaviors, including adherence to offloading and regulation of weight-bearing activity. Additionally, data collected by the Sensoria Core is transmitted to a secure cloud platform, allowing clinicians to monitor adherence, daily step counts, and cadence, thus supporting personalized care plans and education [12,23,24].

To evaluate the accuracy of real-time and remote monitoring of adherence and locomotion metrics (steps and cadence), we integrated the sensor core into two distinct offloading devices: the Foot Defender (Defender Ops, Miami, FL, USA) and the DH Walker by Össur (Formfit^®^ Walker, Össur, Reykjavík, Iceland). Both devices are designed to reduce plantar pressure and support wound healing, yet they differ in structural and material composition. The Foot Defender is a mid-calf boot specifically engineered for DFU management, incorporating a semi-rigid anterior and full-length posterior ankle–foot orthosis (AFO) to redistribute plantar pressure, reportedly reducing it by up to 50% compared to standard boots. It also features a proprietary Absorbium™ viscoelastic polymer insole (Defender Ops, Miami, FL, USA) for enhanced shock absorption, antimicrobial and moisture-wicking linings, and a pneumatic fit system with adjustable air bladders to improve comfort and fit. The design further emphasizes ease of use and aesthetics to support patient compliance. In contrast, the DH Walker by Össur is a knee-level walker-style boot suitable for either foot, with a wide low-profile rocker bottom to promote smooth gait and improved stability. It is designed to accommodate bulky dressings and includes the Active Offloading Insole; however, it does not incorporate the same advanced materials or design features as the Foot Defender.

**Figure 1:**
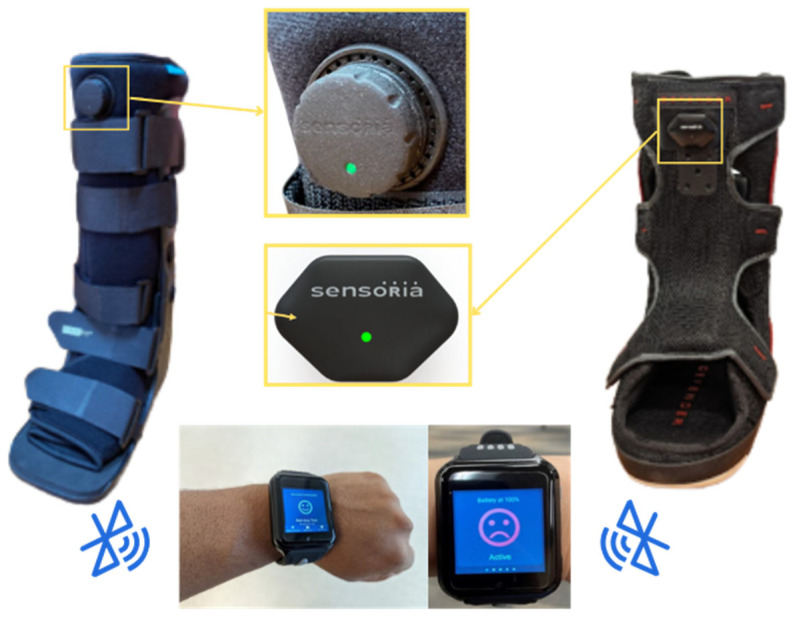
**SmartBoot system with two offloading boots:** The SmartBoot system was evaluated using two offloading boots, DH Walker by Össur (left) and Foot Defender (right), each integrated with the Sensoria Core and paired with a smartwatch. The watch functions as a local hub that processes data in real time and serves as a user interface that provides visual adherence feedback (happy/sad face), displays step counts with and without offloading, and enables cellular data transfer to the cloud. This data supports a remote patient-monitoring portal to assist wound-care specialists in personalizing education and care plans based on adherence trends, changes in gait speed (as a proxy for frailty or muscle loss), and identification of excessive weight-bearing activities that may delay DFU healing.

Both offloading boots were integrated with the same IMU sensor (Sensoria Core, Sensoria Health Inc., Redmond, WA, USA), and the same smartwatch was used to collect data, as shown in Figure 1.

### 2.2. Experimental Protocol and Assessments

**Figure 2:**
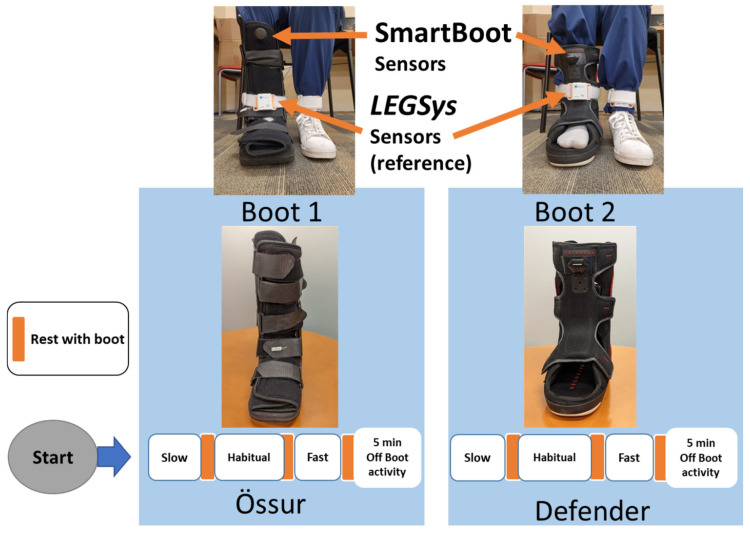
**Experimental setup and protocol**: Participants were asked to wear two standard offloading devices (DH Walker by Össur and Foot Defender) in random order, each instrumented with SmartBoot sensors (Sensoria, Redmond, WA, USA). The protocol included walking at slow, habitual, and fast speeds, followed by 5 min of off-boot activities. A validated wearable gait analyzer (LEGSys, BioSensics LLC, Newton, MA, USA) was used to measure cadence during each walking bout for comparison. Step counts and durations of on-boot and off-boot activities were also recorded by research staff as reference measures. Adequate rest periods were provided between activities to ensure clear distinction among task phases.

Individuals with DFU are advised to wear the offloading boot at all times, including while sitting or standing, as harmful plantar pressure can persist even in non-ambulatory positions. Although unprotected walking carries the greatest risk, prolonged pressure during sitting or standing should not be underestimated [8,19]. We have previously validated the SmartBoot’s ability to detect off-boot activities, including seated and standing conditions without the boot [11]. The primary aim of this study is to assess the algorithm’s accuracy in remotely monitoring walking activity regardless of the type of offloading device used and to report clinically relevant metrics, specifically protected versus unprotected steps and cadence, with the latter serving as a surrogate for frailty-related slowness. The validation study protocol (Figure 2) involved predetermined walking assessments, during which healthy participants, free of DFU or associated risk, wore each offloading boot (Foot Defender and DH Walker by Össur) in random order, while walking at varying speeds (slow, self-selected or habitual, and fast). Each speed condition involved 25 strides (50 steps) and was repeated twice per device, with 1 min rest periods between the trials and assessments, resulting in a cumulative duration of about 15 min. Five-minute physical activities, including walking without the boot, were also conducted to assess adherence. Real-time step count, cadence, and walking speed were concurrently measured using the SmartBoot and were compared with a validated wearable gait analyzer system (LEGSys, BioSensics LLC, Newton, MA, USA) [25,26,27]. The exclusive inclusion of healthy individuals was an ethical safeguard, as intentionally inducing unprotected walking in individuals with or at risk of DFU could exacerbate wounds and pose significant harm.

To assess participants’ perceptions of SmartBoot acceptability, including perceived usefulness (the degree to which the SmartBoot enhances DFU management), perceived ease of use (the extent to which the use of SmartBoot is user-friendly), attitude toward use (the individual’s positive or negative feelings about using the SmartBoot), and behavioral intention to use (the likelihood of continued or future use), we administered a 19-item Technology Acceptance Model (TAM) survey (Table 1) adapted from Finco et al. [22] to both healthy individuals and participants with DFU. Each TAM item was rated on a 5-point Likert scale (1 = strongly disagree to 5 = strongly agree). For each TAM domain, the mean score across relevant items was calculated and scaled from 20 to 100 by multiplying by 20, with higher scores indicating greater acceptability. To capture insights from actual end users, TAM responses were also collected from an independent cohort of 81 DFU patients who had used the SmartBoot as part of their wound care.

### 2.3. Participants

Healthy, ambulatory participants without diabetic foot ulcers (DFUs) or associated risk were recruited from Baylor College of Medicine (Houston, TX, USA; IRB protocol: H-40765), while patients with DFUs were enrolled from the University of Southern California (Los Angeles, CA, USA) as part of an ongoing clinical trial evaluating the SmartBoot’s effectiveness in wound healing (ClinicalTrials.gov Identifier: NCT04460573; IRB protocol: HS-20-00526). Inclusion criteria for DFU patients included being 18 years or older, having an active DFU requiring offloading, and the ability to provide written informed consent. Exclusion criteria included wounds present for over a year, uncontrolled glycemia (HbA1c > 12%), major vascular conditions requiring revascularization during the study period, ulcers involving bone or tendon, non-diabetic ulcers, inability or unwillingness to attend scheduled clinic visits, and full-time wheelchair confinement. Written informed consent was obtained from all participants prior to study initiation.

### 2.4. Data and Statistical Analysis

The SmartBoot enables the remote measurement of daily steps and cadence, with data accessible via its patient-monitoring portal or smartwatch interface, typically with a delay of less than 5 s. To validate the accuracy of SmartBoot-reported values, we used a validated gait analysis system (LEGSys, BioSensics LLC, Newton, MA, USA). Due to potential synchronization challenges, such as cloud transmission delays, we compared total step count and average cadence at the level of individual walking bouts rather than using continuous time-aligned data. The testing protocol included distinct walking bouts at various velocities (habitual, slow, and fast), with sufficient rest intervals between bouts to ensure consistent and comparable data capture across both systems. Accuracy was assessed using Bland–Altman analysis, which quantified agreement between the SmartBoot and LEGSys by calculating limits of agreement (LOAs). This method allowed for an evaluation of both bias (mean difference) and precision (standard deviation of the difference) between the two systems [28]. Additionally, bias and precision were expressed as a percentage relative to the range of cadence value.

(1) $$Bias\;(\%)=\left(\frac{Mean\;difference\;between\;boots\;and\;Legsys\;sensors}{{Range\;of\;parameter\;\left(Max-Min\right)}_{LegSys}}\right)\times 100$$

To assess the accuracy of the SmartBoot’s real-time adherence reporting and step count, an independent observer manually recorded all events during which the boot was worn (“Boot On”) or removed (“Boot Off”), along with the corresponding step counts. Specifically, the experimenter manually recorded the start and end times of each walking assessment using a stopwatch and observation logs, noting when the participant donned or removed the boot. Simultaneously, the SmartBoot system autonomously recorded “Boot On” and “Boot Off” events with precise timestamps. The time segments derived from the experimenter’s records were used as the reference standard. Accuracy was assessed using sensitivity, specificity, and overall accuracy, as calculated in Equations (2)–(4). True positives were defined as time segments and step events where the smartwatch correctly reported “Boot On” while the participant was wearing the boot, as verified by the observer’s notes. False negatives occurred when the smartwatch reported “Boot Off” during periods of confirmed boot wear. True negatives reflected correct identification of “Boot Off” when the boot was not worn, and false positives referred to time segments and step events where the smartwatch incorrectly reported “Boot On” despite the observer’s documentation indicating that the boot was not in use.

(2) $$Sensitivity=\frac{True\;positive}{True\;positive+False\;negative}\times 100(\%)$$

(3) $$Specificity=\frac{True\;negative}{True\;negative+False\;positive}\times 100\left(\%\right)$$

(4) $$Accuracy=\frac{True\;positive+True\;negative}{True\;positive+True\;negative+False\;positive+False\;negative}\times 100(\%)$$

To evaluate the accuracy of the SmartBoot’s real-time step-count reporting, the percentage error relative to the observer-recorded step count was calculated as shown in Equation (5).

(5) $$Percentage\;error\;of\;step\;counts=\frac{Step\;counts\;from\;SmartBoot-Observer\;counted\;step\;counts}{Step\;counts\;from\;SmartBoot}\times 100(\%)$$

To compare perspectives on SmartBoot acceptability between the Foot Defender and DH Walker by Össur offloading types, as well as between healthy individuals and participants with DFU, the mean and standard deviation of responses for each TAM category were reported. Additionally, differences in perceived usefulness, ease of use, behavioral intention to use, and attitude toward use between the two offloading types were analyzed, with effect sizes calculated using Cohen’s d.

### 2.5. Sample Size

This is a secondary analysis of an ongoing clinical trial (ClinicalTrials.gov Identifier: NCT04460573). The primary aim of the parent study is to evaluate the effectiveness of the SmartBoot in improving wound-healing outcomes, with a target enrollment of 210 individuals with DFU to detect a clinically meaningful increase (>10%) in healing rates compared to conventional offloading boots. In this secondary study, our focus is on validating the accuracy of the SmartBoot in remotely monitoring and reporting key parameters, such as adherence, step count, and cadence, and assessing its perceived acceptability. For the validation component, we recruited a convenience sample of 12 healthy individuals. To assess perceived acceptability, we enrolled 81 patients with DFU. While this sample size is adequate for the aims of this analysis, it may be underpowered for between-group comparisons or for evaluating the influence of sex and other potential confounders.

## 3. Results

Twelve participants (30 ± 4.01 years; 58% male) were eligible to participate in this study, and TAM scores were collected from eighty-one DFU patients (57.2 ± 12.8 years; 81% male). Their demographics and clinical information are demonstrated in Table 2.

**Figure 3:**
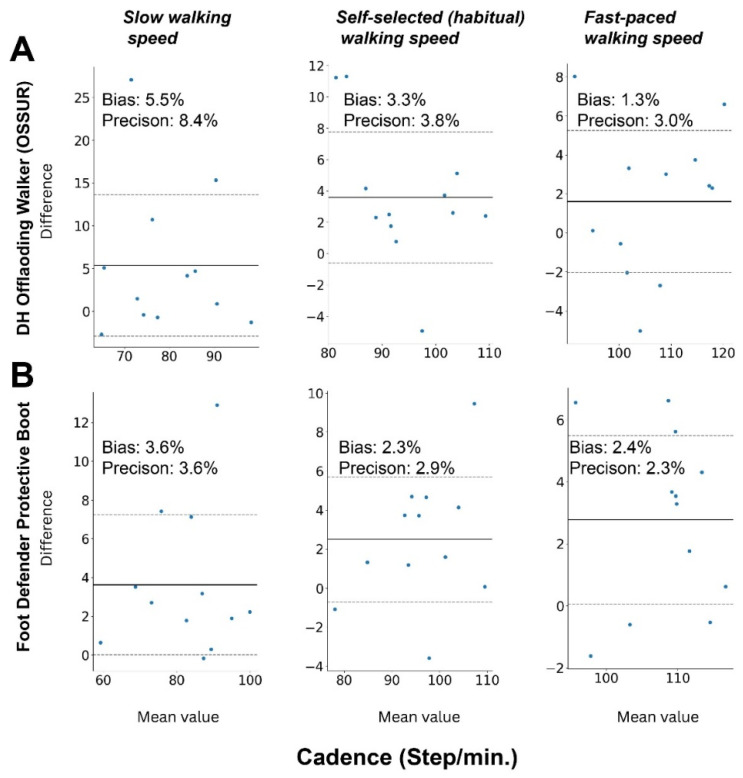
**Bland–Altman analysis for cadence measurement:** The plots show the difference in cadence estimates between SmartBoot and the reference system (LEGSys) versus their mean, illustrating agreement and potential bias across walking speeds for (**A**) the DH Walker by Össur and (**B**) the Foot Defender boot. Cadence is measured in steps per minute (steps/min) across slow, habitual, and fast walking trials.

The Bland–Altman plots demonstrated low bias and high precision across all cadence parameters during slow, habitual, and fast walking tasks for both the Össur (bias: 1.33–5.45%; precision: 3.03–8.41%, with the highest bias and lowest precision observed during slow walking) and Defender (bias: 2.27–3.63%; precision: 2.33–3.62%, with the largest bias and lowest precision also observed during slow walking) boot conditions (Figure 3).

According to the results, measurement of cadence showed significant agreement for both SmartBoots on each walking speed.

Table 3 shows the results of step-count errors from the walking assessments. The results demonstrated that the Foot Defender had less errors than the Össur, especially for fast walking assessment. The gait speeds measured by LEGSys are also mentioned in the table.

The results of the sensitivity, specificity, and accuracy of adherence to boot use are reported in Table 4. The accuracy was measured as 96% and 97% for the Össur and Foot Defender, respectively.

**Figure 4:**
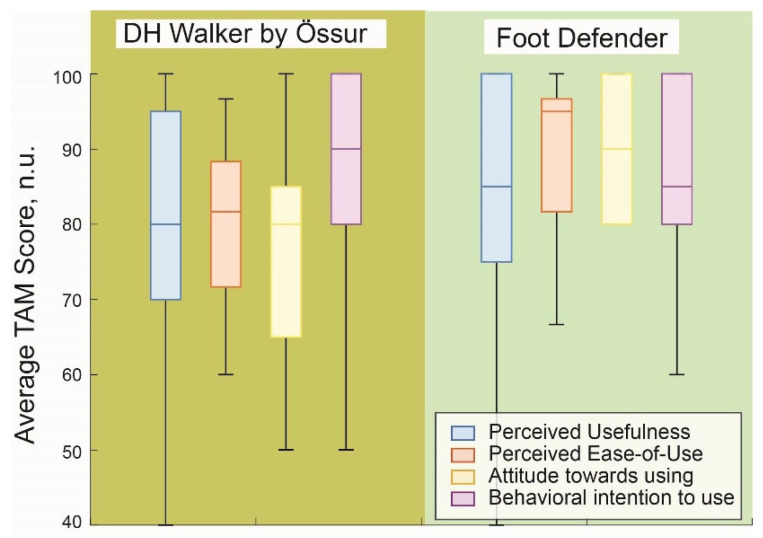
**Average TAM Scores by category for two offloading boots in healthy participants.** This figure presents the average scores (range: 20–100; no units) for four categories of the TAM survey: perceived usefulness, perceived ease of use, attitude toward use, and behavioral intention to use. Scores were collected from healthy participants, evaluating two offloading boots: the DH Walker by Össur and the Foot Defender. A score above 60 corresponds to “agree” or “strongly agree” on the original Likert scale, indicating high perceived acceptability and strong potential for user adoption. The Foot Defender demonstrated slightly higher ratings across all four TAM categories.

Figure 4 demonstrates the results of TAM from healthy subjects, including perceived usefulness, perceived ease of use, attitude toward use, and intent of use. The results showed that the attitude toward use had the highest effect size (Cohen’s d), calculated as 1.30, while perceived ease of use was 0.78, perceived usefulness was 0.19, and intent to use was 0.06.

**Figure 5:**
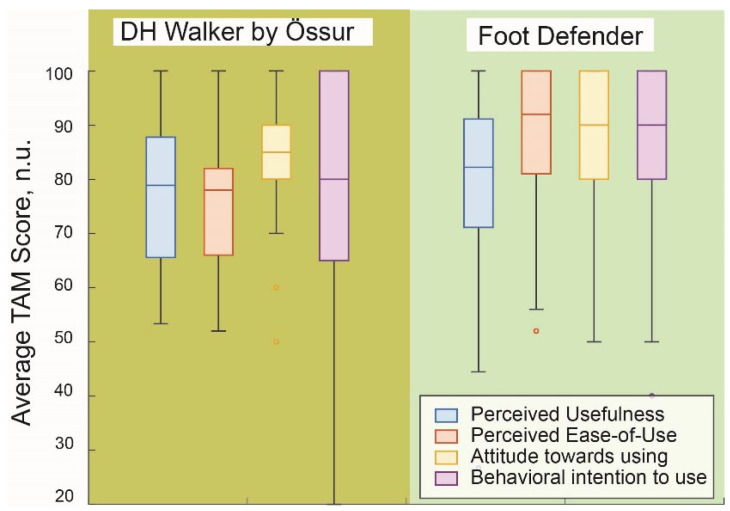
**Average TAM scores by category for two offloading boots in participants with DFU**. Scores above 60 (equivalent to “agree”) indicate high perceived acceptability and potential for adoption and scalability, with slightly higher ratings observed for the Defender boot across all TAM categories. Compared to healthy participants, DFU participants reported slightly lower TAM scores overall; however, perceived usefulness, attitude toward use, and behavioral intention to use remained high, particularly for the Defender boot. A notable difference was observed in perceived ease of use, which was higher for the Defender, likely due to its simpler donning and doffing process compared to the Össur boot.

We also illustrate the TAM results from actual DFU patients in Figure 5 to indicate the acceptance of SmartBoot systems among the DFU patients. The results show that the perceived ease of use had the highest effect size (Cohen’s d), calculated as 0.89, while intent of use was 0.35, attitude toward use was 0.30, and perceived usefulness was 0.07.

## 4. Discussion

This study demonstrates the validity of the SmartBoot system for remotely and objectively monitoring adherence to offloading protocols and capturing key gait-related digital biomarkers, including step count and cadence. The findings indicate that SmartBoot provides accurate estimates of adherence, steps, and cadence across different walking speeds and offloading types. User feedback from both healthy individuals and patients with DFU further supports the system’s usability and acceptability in real-world contexts, suggesting its potential role in complementing standard wound-care practices.

Accurate assessment of adherence to offloading is critical for effective management of DFUs, yet prior studies have shown that self-reported adherence is often unreliable. For example, Ababneh et al. reported a median self-reported adherence of 90%, while objective monitoring using dual-activity monitors revealed actual adherence was only 35%, underscoring a substantial gap between perceived and actual behavior [10]. Although previous objective methods, such as dual-activity monitors and temperature sensors [10,24], have improved accuracy over self-reports, these approaches provide only retrospective data and lack the ability to support real-time intervention. In contrast, the SmartBoot system enables continuous real-time monitoring of adherence, step count, and cadence, and it streams this data remotely. This allows for timely feedback to patients (e.g., via smartwatch notifications), enhancing the potential to reinforce adherence and personalize care strategies [12,24]. Unlike prior research-grade devices limited to in-clinic or offline analysis, SmartBoot integrates adherence monitoring into clinical workflows and supports real-world implementation through secure data transmission; real-time feedback for adherence reinforcement; and proactive management of potentially harmful activity dosage, such as prolonged or continuous walking bouts that may impede wound healing [12,19]. By enabling the monitoring of allowable physical activity (e.g., acceptable cumulative daily steps), SmartBoot may help clinicians personalize offloading strategies. Its integration with remote patient-monitoring platforms and user-friendly design further enhance its clinical utility. Collectively, these features represent a meaningful advancement in the remote management of DFUs, setting SmartBoot apart from traditional sensorized footwear systems in improving DFU care.

Monitoring daily step counts and cadence in individuals with DFUs undergoing offloading treatment is crucial for optimizing wound healing and preventing complications [8,9,29,30]. Excessive mechanical stress from weight-bearing activities can impede DFU healing [8,9]. While offloading devices aim to reduce this stress, patient adherence varies, and overactivity may delay healing [8,9,19,31]. Monitoring daily steps allows clinicians to assess adherence and adjust care plans accordingly. Some studies have found that increased daily steps are associated with slower ulcer healing, though findings are mixed and may depend on individual patient factors [9,19,32].

Cadence, or steps per minute, serves as an indicator of gait speed and overall mobility. Reduced cadence may reflect frailty, which is associated with decreased muscle mass and function. Offloading devices, while beneficial for ulcer healing, can limit mobility and contribute to muscle atrophy [18,33]. Monitoring cadence helps identify patients at risk of developing frailty [23,34,35,36] due to reduced activity levels, enabling timely interventions to maintain mobility and prevent further decline. Incorporating real-time monitoring of daily steps and cadence into DFU management provides valuable insights into patient adherence and mobility, facilitating personalized care strategies to enhance healing outcomes and overall health.

The SmartBoot system demonstrated comparable accuracy in detecting adherence across both the Foot Defender and Össur DH Offloading Walker devices, suggesting that the embedded sensor core can be effectively integrated into various offloading modalities for real-time and remote monitoring. The observed accuracy aligns with findings from a prior study by Park et al. that reported an average sensitivity of 90.6%, specificity of 88.0%, and overall accuracy of 89.3% while using the SmartBoot with the Össur DH Walker [11]. However, a slight improvement in the estimation of cadence and step counts was observed when using the Foot Defender. This difference may be attributed to the sensor’s placement; in the Össur DH Walker, the sensor was positioned beneath the fastener Velcro, potentially introducing motion artifacts, especially during faster walking speeds. These findings underscore the importance of considering sensor placement in optimizing the accuracy of gait parameter measurements. Additionally, in this study, specificity was relatively lower than sensitivity, indicating that the boot was occasionally reported as “on” when it was, in fact, “off.” This discrepancy may be attributed to delays in data transmission between the boot and the smartwatch, latency in data visualization and cloud streaming, and further algorithmic delays in confirming the offloading status. Collectively, these factors may contribute to a lag in accurately detecting the exact moment the boot is removed.

This study found that participants walked more slowly while wearing the Össur DH Offloading Walker compared to the Foot Defender boot across all tested gait speeds. This observation aligns with TAM results, indicating that both healthy individuals and patients with DFUs perceived the Foot Defender as more comfortable and user-friendly. The slower walking speed associated with the Össur DH Walker may be attributed to its greater strut height and weight, which can impede natural gait mechanics and increase the physical effort required for ambulation [37]. These findings are consistent with prior research demonstrating that knee-high offloading devices can reduce gait speed and ankle motion, potentially leading to decreased comfort and adherence [7,9,17,37,38,39]. In contrast, ankle-high offloading devices have been associated with improved comfort and gait parameters, which may enhance patient compliance and, consequently, wound-healing outcomes. Therefore, the design characteristics of offloading devices, including height and weight, are critical factors influencing patient mobility and should be carefully considered to optimize both therapeutic efficacy and user experience.

Beyond diabetic foot syndrome, the SmartBoot’s capabilities in real-time monitoring of adherence, step count, and cadence may have broader clinical applications. These include conditions such as Charcot neuroarthropathy and neurodegenerative disorders like Parkinson’s disease, where gait monitoring and protective-device adherence are critical. Additionally, post-stroke rehabilitation, frailty management in older adults, and postoperative recovery following orthopedic procedures may benefit from such technologies. In these populations, remote feedback could help improve adherence to prescribed interventions, mitigate fall risk, and support early detection of functional decline [40,41,42,43,44].

This study has several limitations that may affect the generalizability of its findings. To validate the SmartBoot’s capability to estimate adherence and gait metrics across various walking conditions, we exclusively recruited healthy participants without DFUs or associated risks. This selection was an ethical safeguard, as inducing unprotected walking in individuals with or at risk of DFUs could exacerbate wounds and pose significant harm. While we anticipate that this limitation does not substantially impact the accuracy of adherence, step count, and cadence estimations, caution is warranted when generalizing these results to the DFU patient population, whose gait patterns may differ from those of healthy individuals. To approximate the walking patterns of DFU patients, participants were instructed to walk at slower speeds, aligning with previously reported walking speeds among DFU patients and those at risk [45,46]. Additionally, the study was conducted under controlled and supervised conditions, relying on observer documentation to estimate adherence due to the absence of validated commercial solutions for unsupervised adherence monitoring. In this study, we were unable to directly measure system performance metrics such as jitter, latency, or execution time, nor were we able to assess technical specifications like power consumption. However, our estimates of sensitivity and specificity inherently accounted for these factors. Importantly, minor delays, in the order of a few seconds, are unlikely to affect clinical utility, as the system is designed to notify patients at the onset of unprotected walking and to provide clinicians with cumulative activity data during routine follow-up visits. Furthermore, in comparing adherence results between self-reports and SmartBoot data, we did not account for potential delays caused by data transmission between the boot and smartwatch, as well as the visualization of data on the watch and stream data to cloud. These factors may contribute to increased errors, particularly affecting specificity. Future studies should aim to compare the accuracy of the proposed solution against self-reported adherence or other modalities during daily activities and investigate its association with wound-healing outcomes.

## 5. Conclusions

In conclusion, this study validated the SmartBoot system as a reliable and acceptable solution for real-time remote monitoring of adherence to offloading protocols and gait-related metrics, including step count and cadence. The system demonstrated consistent accuracy across different walking speeds and offloading modalities, with user feedback supporting its usability in both healthy individuals and patients with diabetic foot ulcers. These findings underscore the potential of SmartBoot to support personalized and continuous care in DFU management and warrant further investigation in real-world, unsupervised settings to evaluate its impact on clinical outcomes.

## Figures and Tables

**Table 1:** **Technology Acceptance Model (TAM) questionnaire for evaluating acceptability and usability.** Perceived acceptability and usability were assessed using a 19-item questionnaire based on the Technology Acceptance Model (TAM), adapted from the study by Finco et al. [22]. The items were grouped into four categories: perceived usefulness, perceived ease of use, attitude toward use, and behavioral intention to use. Each item was rated on a 5-point Likert scale (1 = strongly disagree to 5 = strongly agree).

Categories	TAM Questions
**Perceived usefulness**	Using the smart boot can improve my quality of life
	The smart boot helped me in doing my daily activities
	The smart boot helped me follow the doctor’s instructions
	The smart boot encouraged me to monitor how much I walk
	The design of the smart boot made me want to wear it longer
	Interacting with the boot through the smart watch was helpful to keep me on track
	It is beneficial for the doctor to be able to monitor my activity through the smart boot
	The alerts on the smart watch were easy to understand
	The number of alerts I received were just about right
**Perceived ease of use**	The smart boot was too intrusive
	Learning how to use the smart boot was easy
	Using the smart boot is easy
	The smart boot is easy to take on and off
	The smart boot looks good
	The smart boot is comfortable
**Attitude toward using**	I like using the smart boot
	I think the smart boot is a good idea
**Behavioral intention to use**	I would like to use the smart boot in the future
	I would recommend the smart boot to my friends

**Table 2:** Participants’ demographic information.

	Healthy Subjects (n = 12)	DFU Patients (n = 81)
Age, years	30 ± 4.01	57.2 ± 12.8
Gender (male), %	58%	81%
Ethnicity (Hispanic), %	25%	53%
BMI	31.4 ± 11.9	33.1 ± 9.9

**Table 3:** Absolute and relative step-count error measured across each gait-speed protocol.

Gait Protocol	Slow		Habitual		Fast	
Average of #steps (DH Walker by Össur)	51.8 ± 6.3		51.6 ± 4.1		46.8 ± 7.8	
Average of #steps (Foot Defender)	49.5 ± 2.9		46.8 ± 7		48.1 ± 7.3	
	Step count error * *(steps / %)*	Speed (m/s)	Step count error * *(steps / %)*	Speed (m/s)	Step count error * *(steps / %)*	Speed (m/s)
DH Walker by Össur	1.83 (3.67)	0.50	1.58 (3.17)	0.81	3.17 (6.33)	1.10
Foot Defender	0.50 (1)	0.56	0.67 (1.5)	0.89	0.17 (0.5)	1.18

* The step count error is presented as absolute error (abs) with units of steps, and relative error (rel) with units of %.

**Table 4:** **Accuracy, sensitivity, and specificity of adherence detection for two offloading devices.** This table summarizes the performance of the SmartBoot system in detecting adherence to prescribed offloading use for two different offloading devices. Accuracy, sensitivity, and specificity were calculated based on comparison with annotated ground-truth data (recorded by an observer). Adherence was defined as wearing the offloading device, regardless of the type of activity performed.

	Accuracy (%)	Sensitivity (%)	Specificity (%)
DH Walker by Össur	96	99	86
Foot Defender	97	99	88

## Data Availability

Data is available upon request by contacting the corresponding author.
